# Optimization and Preparation of Polysaccharide–Protamine Microspheres with Enhanced Hemostatic and Antibacterial Properties for Wound Healing

**DOI:** 10.3390/md23040160

**Published:** 2025-04-06

**Authors:** Danling Mei, Feifan Cheng, Yifan Li, Suzhen Zhang, Xueqin Zhao, Yanyan Zhao

**Affiliations:** College of Life Sciences and Medicine, Zhejiang Sci-Tech University, Hangzhou 310018, China

**Keywords:** protamine, carboxymethyl starch, hydroxypropyl trimethyl ammonium chloride chitosan, layer-by-layer self-assembly

## Abstract

This study employs layer-by-layer self-assembly technology to develop novel antibacterial hemostatic microspheres to tackle significant blood loss and related complications resulting from accidents, surgeries, and natural disasters. By measuring the zeta potential and particle size of protamine, carboxymethyl starch (CMS), and hydroxypropyl trimethyl ammonium chloride chitosan (HACC), the optimal assembly conditions were determined. The optimal pH for the monolayer assembly is 3.0, with a CMS concentration of 3 mg/mL and a mass ratio of 1:4 between protamine and CMS, and the assembly process lasts for 2 h. The optimal assembly conditions for the bilayer assembly are a pH of 4.0, an HACC concentration of 1 mg/mL, and a mass ratio of the one-layer assembly to HACC of 1:2. The performance of the assembled microspheres was analyzed via antibacterial and coagulation experiments, revealing excellent antibacterial and coagulation effects, with inhibition rates against *Escherichia coli* and *Bacillus subtilis* both exceeding 99%, and a coagulation index of 0%. Additionally, the bilayer assembled microspheres also exhibited strong adsorption capacity and good biocompatibility. In summary, this study provides important scientific evidence for the development of new hemostatic materials, demonstrating significant clinical application potential.

## 1. Introduction

Accidents, trauma resulting from accidents, surgical procedures, and natural disasters can lead to severe blood loss, which is considered one of the leading causes of death in contemporary human society [1]. Excessive blood loss can result in shock, organ damage, or even death. The application of hemostatic dressings is among the most effective strategies to mitigate such outcomes. However, current traditional hemostatic devices and medications carry risks such as tissue necrosis and thrombosis [2]. In recent years, researchers have developed various new types of biomaterials, including hemostatic sponges, hemostatic nanofiber membranes, novel hemostatic hydrogels, and new hemostatic microspheres [3]. Compared to traditional materials, these new materials can incorporate clotting agents like thrombin to actively promote hemostasis or provide advantages in terms of suitability for various wound types and biodegradability [4].

Late-stage infections are common complications during treatment [5]. Beyond hemostatic efficacy, antimicrobial performance is crucial for preventing infections and wound deterioration. Many wound dressings incorporate complexes with antibiotics, peptides, or metals to achieve the desired antimicrobial properties [6,7]. However, antibiotic resistance and metal toxicity limit their practical use. Furthermore, the high cost of these methods restricts their widespread application [8]. Therefore, there is an urgent need for affordable wound dressings with broad-spectrum antimicrobial activity and good biocompatibility.

Protamine is an alkaline cationic polypeptide primarily found in the mature testicular tissues of fish and other species, possessing clotting activity [9]. It can activate fibrinogen receptors, promote α-granule release, and directly enhance platelet aggregation, improving clotting efficacy. Additionally, it exhibits specific clotting effects through heparin binding [10,11,12]. Apart from its clotting properties, protamine demonstrates excellent biocompatibility and non-toxicity, making it widely used in the medical field [13,14].

Chitin is a natural high-molecular-weight compound widely present in the shells of crustaceans and arthropods. A commonly used and important derivative of chitin is obtained by removing the N-acetyl groups from glucosamine, known as chitosan. Hydroxypropyl trimethyl ammonium chloride chitosan (HACC) is formed by introducing hydroxypropyl trimethyl ammonium chloride onto chitosan, rendering it highly water-soluble and versatile due to the incorporation of strongly hydrophilic groups [15]. Previous studies have indicated that HACC exhibits broad-spectrum antibacterial properties, serving as an antibacterial agent by adsorbing to bacterial surfaces via its inherent positive charge, hindering the absorption and transport of nutrients or causing a leakage of essential substances such as cellular enzymes, thereby exerting antibacterial effects [16]. HACC plays a significant role in coagulation, antibacterial properties, biocompatibility, and degradability, making it a valuable material for preparing coagulation microspheres [17].

In recent years, the layer-by-layer self-assembly technology (LbL) of molecular aggregates has attracted increasing attention due to the spontaneous self-assembly of polymer or polyelectrolyte groups in solution on a substrate, forming structurally complete, stable entities with specific functions [18]. LbL assembly is a method of alternating the assembly of polymers or polyelectrolytes using electrostatic attraction between opposite charges. This technique is known for its simplicity, mild preparation conditions, efficient maintenance of biological activity, and precise control over microstructure, and it has found widespread applications in the medical and chemical industries [19].

Carboxymethyl starch sodium (CMS) is derived from natural plant polysaccharide starch and can dissolve in cold water to form an anionic polyelectrolyte ether [20]. In the pharmaceutical industry, CMS is often utilized in the form of hydrogels or nanoparticles for drug delivery due to its excellent biocompatibility, biodegradability, and water absorption and swelling properties [21,22]. In this study, CMS was used as a stabilizer to load protamine. The electrostatic interaction between their surface potentials was utilized to prepare well-dispersed monolayer assemblies. This study investigated the potential, encapsulation efficiency, and biocompatibility of the prepared protamine/CMS monolayer assemblies. Subsequently, protamine/CMS/HACC antimicrobial coagulation microspheres were prepared using the electrostatic adsorption interaction of anionic and cationic ions. Additional experiments optimized conditions such as the pH, temperature, assembly time, and different mass ratios of the assembly materials, followed by characterization and evaluation of the microspheres’ structure and performance. In conclusion, this research offers crucial scientific insights for the development of new hemostatic materials, highlighting their substantial potential for clinical use.

## 2. Results

### 2.1. Effects of pH and Concentration on Electrostatic Properties of Protamine and CMS for Monolayer Microspheres Assembly

**Figure 1:**
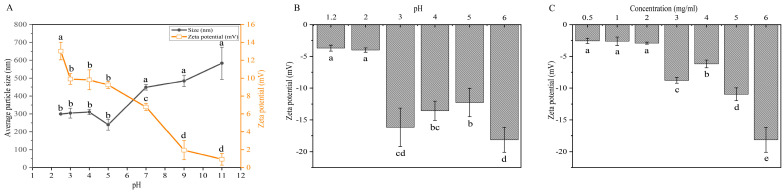
Preparation of composites for monolayer microsphere assembly. (**A**) Influence of pH on protamine and average particle size of protamine. (**B**) Effect of pH on the zeta potential of CMS. (**C**) Effect of CMS concentration on its zeta potential. The data were analyzed using one-way ANOVA. Different letters (a, b, c, d, e) indicate significant differences between groups (*p* < 0.05).

The self-assembly mechanism of the protamine/CMS/HACC composite microspheres relies on the electrostatic interaction between the composite materials. The first step in this process is the assembly of the inner layer monolayer microspheres, which have protamine at their core and are encapsulated in CMS. Due to the smaller diameter and higher electrostatic charge of protamine, it performs better in terms of drug efficacy and assembly efficiency. To achieve optimal self-assembly, adjusting the pH of the protamine solution is crucial [23]. By changing the pH, the surface charge (zeta potential) and average particle size of protamine can be effectively regulated (Figure 1A). At a pH of 11, protamine exhibits a minimal charge and maximum average particle size, indicating that it is close to its isoelectric point. As the pH decreases, the amino groups of protamine bind with more hydrogen ions, leading to electrostatic repulsion between the positively charged protamine molecules. This reduces intermolecular aggregation and enhances the stability of the solution. Notably, when the pH falls below 5, protamine molecules show a higher positive charge and smaller average particle size, providing favorable conditions for electrostatic layer-by-layer self-assembly applications (Figure 1A).

CMS serves as the intermediate layer of the bilayer microspheres and functions as a binder; hence, it should carry a charge opposite to that of protamine. Here, we explore the effects of different pH conditions on the charge of CMS. As shown in Figure 1B, CMS carries a negative charge below pH 6, and at pH 3, CMS exhibits a peak zeta potential, making pH 3 the optimal pH for preparing the reaction solution for monolayer assembly.

Different concentrations of CMS solutions can affect their electrostatic charge characteristics. We investigated the impact of various CMS solution concentrations on the electrostatic properties of CMS under pH 3 conditions (Figure 1C). The results indicate that as the CMS concentration increases, the zeta potential shows a significant upward trend. When the CMS concentration exceeds 3 mg/mL, the zeta potential stabilizes at a relatively high level, making it suitable for subsequent monolayer assembly preparation. However, it is important to note that higher concentrations may accelerate the precipitation of CMS after ultrasonic treatment. Therefore, we selected 3 mg/mL as the optimal concentration condition for CMS assembly preparation.

### 2.2. Effects of Mass Ratio, Assembly Time, and Temperature on Encapsulation Efficiency of Protamine Monolayer Assembly

**Figure 2:**
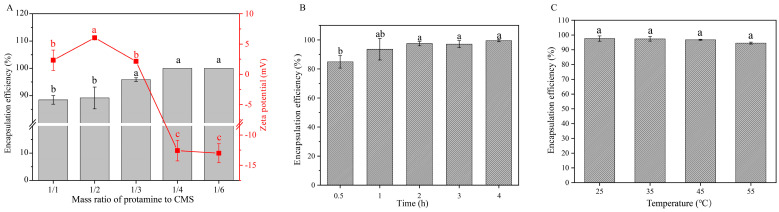
Optimization of monolayer assembly. (**A**) Effect of the mass ratio of protamine to CMS on encapsulation efficiency and zeta potential, respectively. (**B**) Effect of assembly time on protamine encapsulation efficiency. (**C**) Effect of assembly temperature on protamine encapsulation efficiency. The data were analyzed using one-way ANOVA. Different letters (a, b, c) indicate significant differences between groups (*p* < 0.05).

The different mass ratios of protamine to CMS can affect the encapsulation efficiency of protamine. The results indicate that when the mass ratio is greater than 1:4, the monolayer assembly exhibits lower positive charge, which may be attributed to the high positive charge of protamine masking the negative charge of CMS (Figure 2A). In contrast, when the mass ratio is less than or equal to 1:4, the assembly shows higher negative charge, and the encapsulation efficiency reaches 100% (Figure 2A). However, once the encapsulation efficiency reaches 100%, further decreases in the mass ratio inevitably lead to a reduction in drug loading capacity. Therefore, a mass ratio of 1:4 between protamine and CMS can be considered the optimal condition for preparing monolayer assemblies to achieve the best encapsulation efficiency and appropriate drug loading capacity.

The assembly time is critical for the formation of the monolayer components. As the assembly time increases from 0.5 h to 2 h, the encapsulation efficiency of protamine gradually improves. Beyond 2 h, the encapsulation rate reaches its maximum value (Figure 2B). To optimize efficiency and minimize time consumption, an assembly duration of 2 h is recommended for preparing the monolayer assembly.

We also analyzed the impact of assembly temperature on encapsulation efficiency. As shown in Figure 2C, when the temperature increased from room temperature (25 °C) to 55 °C, there was no significant change in the encapsulation efficiency of the protamine. This result indicates that variations in temperature do not significantly affect encapsulation efficiency. Therefore, room temperature (25 °C) is determined to be the optimal condition for preparing the monolayer assembly to ensure the best encapsulation results.

### 2.3. Effects of pH and HACC Concentration on the Preparation of Bilayer Assemblies

**Figure 3:**
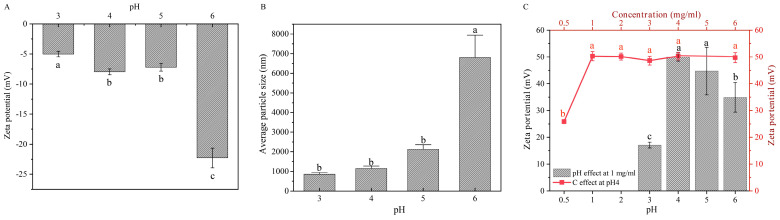
The preparation of composites for bilayer microsphere assembly. (**A**) Effect of pH on zeta potential of monolayer assemblies. (**B**) Influence of pH on average particle size of monolayer assemblies. (**C**) Effect of pH and HACC concentration on the zeta potential of HACC. The data were analyzed using one-way ANOVA. Different letters (a, b, c) indicate significant differences between groups (*p* < 0.05).

The second step in the assembly of bilayer microspheres involves combining the monolayer composites with hydroxypropyl trimethyl ammonium chitosan (HACC) through electrostatic interactions. First, we examined the average particle size and zeta potential of the monolayer composites under different pH conditions (Figure 3A,B). The results reveal that when the pH value ranges from 3 to 6, the monolayer assemblies exhibit a negative charge, and both the charge density and particle size increase as the pH rises. For instance, in the pH range of 3 to 4, the particle size of the monolayer assemblies is relatively small, while it significantly increases in the pH range of 5 to 6 (Figure 3B).

Additionally, we studied the charge properties of HACC under varying pH conditions. The results show that within the pH range of 3 to 6, HACC consistently exhibits a high positive charge, and its charge density follows a trend of initially increasing and then decreasing with rising pH, reaching a maximum at pH = 4 (Figure 3C). By integrating the zeta potential and particle size data of the monolayer assemblies from Figure 3A,B, it can be inferred that at pH = 4, HACC and the monolayer assemblies possess opposite charges with significant charge densities. At this pH, the particle size of the monolayer assemblies is relatively small, which may indicate higher stability. Therefore, this pH condition can be considered an ideal choice for the preparation of bilayer assemblies.

We also examined the influence of HACC concentration on the charge density. As shown in the figure, when the HACC concentration increases from 0.5 mg/mL to 1 mg/mL, the charge density exhibits a significant increasing trend, and then stabilizes around 50 mV (Figure 3C). Therefore, concentrations above 1 mg/mL can be used as the HACC concentration conditions for the subsequent preparation of the bilayer assembly. However, as the HACC concentration increases, the viscosity of the solution also rises, complicating the stirring process and significantly impacting the recovery efficiency. Therefore, a concentration of 1 mg/mL was ultimately designated as the optimal HACC concentration for the preparation of the bilayer assembly.

### 2.4. Effects of HACC Concentration and Mass Ratio of (Protamine-CMS)/HACC on Encapsulation Efficiency of Protamine Bilayer Assembly

**Figure 4:**
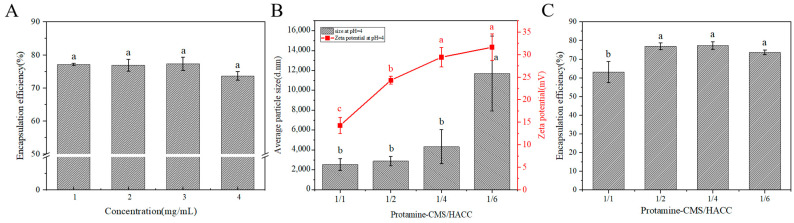
Assembly of the monolayer composite with HACC. (**A**) Effect of HACC concentration on protamine encapsulation efficiency. (**B**) The effect of monolayer assembly (Pro-CMS)/HACC mass ratio on the average particle size and zeta potential of bilayer assemblies. (**C**) The effect of various mass ratios of monolayer assembly (Protamine-CMS)/HACC on protamine encapsulation efficiency. The data were analyzed using one-way ANOVA. Different letters (a, b, c) indicate significant differences between groups (*p* < 0.05).

We investigated the influence of HACC concentration on the encapsulation efficiency of protamine. The results indicate that increasing the HACC concentration from 1 mg/mL to 4 mg/mL has no significant impact on the encapsulation efficiency of protamine (Figure 4A). Considering that higher concentrations of HACC solutions exhibit increased viscosity, which may affect stirring, a concentration of 1 mg/mL was chosen as the condition for preparing the bilayer assembly.

The effect of various mass ratios of monolayer assembly (Protamine-CMS)/HACC on protamine encapsulation efficiency was also investigated. The bilayer assemblies prepared with various mass ratios of the monolayer assembly to HACC exhibit high positive charge characteristics (Figure 4B). When the mass ratio is less than 1/2, the zeta potential remains above 25 mV, which meets the criteria for preparing bilayer assemblies (Figure 4B). However, at mass ratios below 1/6, substantial aggregation can cause a significant increase in particle size (Figure 4B). Conversely, when the mass ratio exceeds 1/4, the particle size is relatively small (Figure 4B), and the assemblies are more tightly bound, making them suitable for bilayer assembly preparation as well. Additionally, as indicated in Figure 4C, the encapsulation efficiency of protamine is approximately 75% when the mass ratio is less than or equal to 1/2. However, a smaller mass ratio correlates with a reduced drug loading capacity of protamine. Therefore, a mass ratio of 1/2 is ultimately determined to be the optimal condition for preparing the bilayer assembly.

### 2.5. Characterization of Assembled Bilayer Microspheres

**Figure 5:**
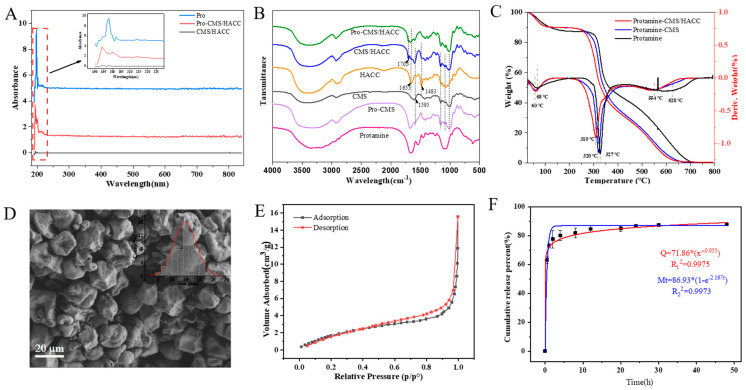
Characterization of assembled bilayer microspheres. (**A**) Ultraviolet full-wavelength scanning of protamine, blank (CMS/HACC) assemblies, and bilayer (Protamine-CMS/HACC) assemblies. (**B**) FTIR spectra of polysaccharide–protamine microspheres (Pro-CMS/HACC), blank (CMS/HACC) assemblies, HACC, CMS, monolayer assembled product of protamine (Pro-CMS) and protamine. (**C**) TG and corresponding DTG curves for bilayer (Protamine-CMS/HACC) assemblies, monolayer assembled product of protamine and protamine. (**D**) Analysis of the morphology of bilayer assemblies in a pH 4.0 solution using Scanning Electron Microscopy (SEM). (**E**) The adsorption capacity of bilayer assemblies. (**F**) Cumulative release (%) of bilayer (Protamine-CMS/HACC) assemblies in pH 7.4 PBS media at 28 °C.

Ultraviolet full-wavelength scanning was used to assess the successful assembly of bilayer microspheres (Figure 5A). The protamine exhibited a significant maximum absorption peak at 200 nm, while the bilayer assembly also displayed characteristic absorption features of protamine at the same wavelength. In contrast, the blank assembly showed no absorption peaks. These results confirm the successful encapsulation of protamine. FT-IR analysis and TG were performed to confirm the presence and possible interactions of CMS/HACC and protamine. As shown in Figure 5B, the intensive band at 1655 cm^−1^ in the HACC spectrum shifted to 1705 cm^−1^ in the Protamine-CMS/HACC and CMS/HACC spectrum due to the superposition effect of the polyelectrolyte complex, suggesting the presence of inter-molecular interactions between CMS and HACC. Moreover, TG and DTG curves of microspheres shifted to a lower-temperature section, and the maximum weight loss temperature decreased with the increase in the layers (Figure 5C), indicating the interactions of polyelectrolyte layers.

The morphology of bilayer assemblies in a pH 4.0 solution was characterized using Scanning Electron Microscopy (SEM), as shown in Figure 5D. However, some microspheres were observed to aggregate, which may have been related to the viscosity of the CMS and HACC solutions and could potentially have affected the accuracy of particle-size measurements. Additionally, the adsorption capacity of bilayer assemblies was also investigated. As shown in Figure 5E, according to the IUPAC classification, the isotherm profile could be classified as type IV, indicating that the adsorption capacity is significantly enhanced due to capillary condensation [24]. The H4-type hysteresis loop (relative pressure at 0.6–0.9) indicated the presence of abundant narrow mesopores in the aggregates. The BET surface area (25.95 m^2^/g), pore volume (75.43 × 10^−3^ cm^3^/g) and average pore size (19.84 nm) suggest the good adsorption capacity of the cross-linked microspheres, which is beneficial for hemostatic applications.

A PBS environment at pH 7.4 was used to simulate the wound interface for release studies. As shown in Figure 5F, the release of protamine exhibited a trend of rapid release followed by slower release. In the weakly alkaline medium, approximately 63.1% of the protamine was rapidly released within the first 30 min, reaching 73.04% after 1 h. Subsequently, it entered a slow release phase, with the release rate of protamine reaching 86.7% within 24 h and leveling off. Kinetic fitting analysis revealed that the release curve followed a first-order kinetic release model. Considering the polymer drug matrix, Peppas model fitting was also performed, yielding *n* = 0.06, which is less than 0.45, indicating Fickian diffusion release. This suggests that the release process is controlled by the concentration gradient. The release results of protamine demonstrate that a significant portion of the protein can be released within a reasonable timeframe, ensuring that the wound receives the necessary bioactive components. This release characteristic is particularly important for acute medical treatments, as it can provide sufficient protamine in a short period to promote hemostasis and healing.

### 2.6. Antibacterial Efficacy and Coagulation Indices of the Bilayer Assemblies

**Figure 6:**
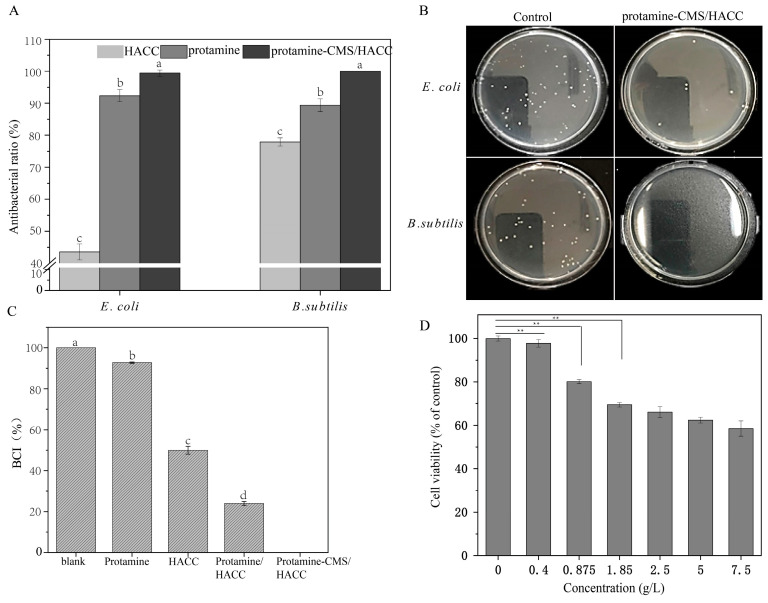
Antibacterial efficacy and coagulation indices of the bilayer assemblies. (**A**,**B**) The antibacterial efficacy of protamine, HACC, and the bilayer assembly. The data were analyzed using one-way ANOVA. Different letters indicate significant differences between groups (*p* < 0.05). (**C**) The coagulation indices of the protamine, HACC, protamine/HACC and bilayer assemblies. The data were analyzed using one-way ANOVA. Different letters indicate significant differences between groups (*p* < 0.05). (**D**) Biocompatibility of the bilayer assemblies. Data were analyzed using a *t*-test; ** indicates a significance level of *p* < 0.01.

The antibacterial efficacy of the bilayer microspheres was tested by antibacterial experiments. The results indicate that protamine, HACC, and the bilayer assembly all exhibit significant antibacterial capabilities (Figure 6A,B). Protamine exhibits significant antibacterial activity against both *E. coli* and *B. subtilis*, with inhibition rates exceeding 85%. HACC also shows considerable antibacterial activity against *B. subtilis*, achieving an inhibition rate of 77.88%. However, HACC has a relatively weak antibacterial effect on *E. coli*, with an inhibition rate of only 43.51%. The bilayer assembly microsphere exhibits excellent antibacterial performance against both bacterial strains, with inhibition rates approaching 100%, providing reliable support for wound treatment.

We analyzed the coagulation indices of protamine, HACC, protamine/HACC and bilayer assemblies. The results indicate that protamine, HACC, and bilayer assemblies all possess coagulation capabilities (Figure 6C). Specifically, the coagulation index of protamine exceeds 90%, suggesting a relatively weak coagulation effect. HACC has a coagulation index of 49.97%, demonstrating better coagulation performance compared to protamine. The coagulation index of the monolayer assembly is 24.02%. Notably, the coagulation index of the bilayer assembly is 0%, significantly lower than that of the protamine/HACC assembly, indicating its excellent coagulation properties. In addition, the biocompatibility of bilayer assemblies was determined by a CCK8 assay in a concentration-dependent manner. As shown in Figure 6D, the cell viability reached 70% and over 80% at tested concentrations of 1.85 mg/mL and 875 μg/mL, respectively, indicating low cytotoxicity for biomedical applications.

## 3. Discussion

With the continuous growth of the demand for modern medical care, especially in the field of trauma treatment, the requirements for hemostatic materials and products are also on the rise. Researchers have begun to explore new hemostatic materials in order to overcome the limitations of traditional methods [21,25]. These new materials are expected not only to possess excellent hemostatic properties but also to exhibit antibacterial characteristics, which can reduce the risk of infection and promote wound healing [26]. In this context, protamine, CMS, and HACC, as new biomaterials, have been widely applied in the medical field due to their excellent biocompatibility, hemostatic properties, and antibacterial characteristics [27,28,29]. They are utilized in various areas, including wound management, drug delivery systems, and tissue engineering, providing significant support for the development of modern medicine.

In a previous study, chitosan and protamine were mixed using an ionic gelation method and subsequently cross-linked with sodium tripolyphosphate (TPP) to prepare chitosan/protein hybrid nanoparticles, which enhanced the antibacterial activity of chitosan nanoparticles against pathogenic *E. coli*, revealing the potential of chitosan/protamine composites in antibacterial applications [30]. This study prepared (Protamine-CMS)/HACC antibacterial hemostatic microspheres through electrostatic adsorption interactions between cations and anions. Protamine, CMS, and HACC are all bio-derived green materials, with no exogenous components introduced. The strategic integration of these materials and the subsequent fabrication of microspheres highlight substantial application potential, effectively addressing the criteria for biocompatibility and functionality. Furthermore, while most reported chitosan-/protein-mixed nanoparticles are in the form of hydrogels, this study opted for a powder form. Powders have broad applicability, suitable not only for film wound dressings but also for effectively addressing various types of wounds, including visceral injuries [31]. Therefore, the antibacterial hemostatic microspheres developed in this study hold significant clinical application potential.

Layer-by-layer self-assembly technology utilizes the natural positive and negative charge characteristics of materials, enabling an efficient and impurity-free self-assembly process [32,33,34]. In our study, we employed an electrostatic self-assembly method, which allows for the layer-by-layer assembly of different components (such as protamine, carboxymethyl starch (CMS), and hydroxypropyl trimethyl ammonium chloride chitosan (HACC)) through electrostatic interactions. This ensures that no exogenous impurities are introduced during the assembly process, helping to maintain the biocompatibility of the materials. By applying multilayer encapsulation to the microspheres, we can significantly enhance their encapsulation efficiency. Initial single-layer microspheres may experience leakage during drug release, while multilayer encapsulation can form a denser structure, greatly improving drug stability and release control [34]. This study assembles protamine with CMS and HACC into bilayer microspheres to leverage the structural advantages, reduce leakage during the drug release process, and ensure that the drug can be released in the desired manner within the specified time, thereby enhancing the therapeutic effect.

In this study, the optimal assembly conditions were determined based on the charge matching of electrostatic interactions and the optimization of assembly efficiency. For the monolayer assembly of protamine with CMS at pH 3.0, protamine carries a positive charge due to its high isoelectric point, approximately 10–12, while CMS carries a negative charge as its carboxyl groups partially ionize under acidic conditions (Figure 1). This charge complementarity enables the two components to efficiently combine through electrostatic interactions, forming a stable monolayer structure. When the mass ratio of protamine to CMS is 1:4, the assembly exhibits a high negative charge, with an encapsulation efficiency reaching 100% (Figure 2A). This indicates that at this mass ratio, the negative charge of CMS is fully utilized, forming strong electrostatic interactions with protamine, thereby enhancing the encapsulation effectiveness. High encapsulation efficiency is crucial for drug release and therapeutic efficacy; therefore, a 1:4 mass ratio is selected as the optimal condition. In the assembly of bilayer microspheres, the process of combining the monolayer composite with HACC through electrostatic interactions demonstrates the importance of pH. Under pH 4.0 conditions, HACC carries a positive charge due to the ionization of its quaternary ammonium groups over a broad pH range, while the protamine/CMS composite remains negatively charged. This charge complementarity ensures that HACC can effectively assemble the second layer on the protamine/CMS single layer. Through a systematic study of the charge characteristics and encapsulation efficiency of the monolayer assembly (protamine-CMS)/HACC at different mass ratios, we found that when the mass ratio is less than or equal to 1:2, the ζ potential remains above 25 mV, indicating that the charge characteristics meet the preparation standards for bilayer assembly, while the encapsulation efficiency of protamine is approximately 75%, ensuring efficient drug loading capacity (Figure 4C). Furthermore, when the mass ratio exceeds 1:4, the particle size is relatively small, leading to a tighter assembly, thus enhancing the applicability of the double-layer assembly (Figure 4B). However, a smaller mass ratio results in a lower drug loading capacity for protamine. Therefore, a 1:2 mass ratio not only effectively balances electrostatic interactions and assembly stability but also ensures high encapsulation efficiency and drug loading capacity, making it the optimal choice for forming bilayer microspheres.

As a cationic polymer, the addition of HACC not only enhances the structural stability of the microspheres but also improves their antibacterial properties [35]. The antibacterial activity of HACC is closely related to its molecular structure, including the degree of substitution, the position of substituents, and the degree of cross-linking [36]. There may be significant differences in these structural parameters among the different forms of HACC produced by different manufacturers, leading to varying antibacterial effects. To evaluate the synergistic effect of our materials, we conducted comparative experiments, which showed that the inhibition rate of *E. coli* and *B. subtilis* was indeed lower when HACC was used alone (Figure 6A). This may be related to the lower degree of substitution or higher degree of cross-linking of the HACC we used. However, when HACC was combined with protamine and CMS to form composite microspheres, its antibacterial activity was significantly enhanced, with an inhibition rate exceeding 99% (Figure 6A). This indicates that the synergistic effect of the composite material plays a crucial role in enhancing antibacterial performance. The composite microspheres exert antibacterial effects through multiple mechanisms, including the electrostatic interaction between the positive charge of HACC and the negative charge of bacterial cell membranes, which disrupts membrane integrity; the antimicrobial peptide properties of protamine further damage the cell membrane, and the enhanced adsorption capability of CMS allows antibacterial components to come into more effective contact with bacteria. Our research findings are consistent with existing reports, indicating that HACC exhibits significant synergistic effects when combined with other antibacterial agents, such as polyhexamethylene biguanide (PHMB) and benzalkonium chloride (BAC) [35,37]. For instance, the use of HACC alone demonstrates limited antibacterial efficacy; however, when combined with PHMB, the HACC-PHMB formulation significantly enhances antibacterial activity, achieving over a 3-log reduction against various pathogens while maintaining high biocompatibility.

Protamine is a cationic protein that can bind to anionic heparin, thereby neutralizing its anticoagulant effect. This allows protamine to be used during surgery to reverse the anticoagulant effects of heparin and help restore normal coagulation function [11]. CMS is widely used in surgical procedures, trauma treatment, and the management of significant bleeding to support blood circulation and coagulation function [29]. In this study, the coagulation index of protamine is over 90% (Figure 6C), indicating a weak coagulation effect. This may mainly function to counteract the anticoagulant action of heparin. The coagulation index of HACC is 49.97% (Figure 6C), showing better coagulation effects than protamine, possibly by absorbing water molecules from the blood, thereby increasing the concentration of blood cells and enhancing coagulation. The coagulation index of the protamine/HACC group is 24.02% (Figure 6A), reflecting a simple additive relationship between the coagulation effects of the two components. The coagulation index of the bilayer assembly is 0% (Figure 6C), significantly lower than that of the protamine/HACC group, indicating excellent coagulation performance. This may be attributed to the increased density of surface pores after the material is prepared into microspheres, enhancing water absorption. In addition, the bilayer-assembled microspheres exhibited strong adsorption capacity (Figure 5E), allowing them to rapidly absorb the liquid components of blood during bleeding and thereby accelerating the hemostatic process. Due to the excellent coagulation performance of the bilayer assembled microspheres, the bilayer assembly can serve as an effective hemostatic material, widely used in surgical procedures, trauma treatment, and emergency care to rapidly control bleeding.

High biocompatibility is an important characteristic that ensures the effectiveness and safety of hemostatic materials [38]. Protamine, CMS, and HACC have all been reported to have good biocompatibility and are widely used in the medical field [13,39,40]. For example, CMS has been used to develop low-cost and biocompatible semi-interpenetrating polymer network (semi-IPN) hydrogels that exhibit optimal synergistic properties, demonstrating good performance in swelling kinetics, thermal analysis, and viscoelastic characteristics, making them suitable as a matrix for drug delivery systems [20]. In a previous study, the cell viability of N2-HACC/CMCS/AMX NPs was approximately 70% at the concentration of 50 μg/mL [41]. In our study, the cell viability of the bilayer assembled microspheres reached 70% and over 80% when the tested concentrations were at 1.85 mg/mL and 875 μg/mL, respectively (Figure 6D). Therefore, the microspheres assembled in this study exhibit improved biocompatibility, enhancing their applicability in the hemostatic field.

Overall, this study provides a scientific basis for the development of novel antibacterial and coagulation materials and opens new avenues for clinical trauma management. As the demand for trauma care continues to rise, these multifunctional antibacterial hemostatic microspheres are expected to play an important role in future medical applications. Future research can further explore the applicability of these materials for different types of wounds and assess their long-term effects in clinical settings, laying a foundation for the broader adoption of novel hemostatic materials.

## 4. Materials and Methods

### 4.1. Materials

Protamine sulfate (Grade X) was purchased from Sigma-Aldrich, St. Louis, Missouri, USA. Carboxymethyl chitosan (CMS) of pharmaceutical grade was obtained from Shanghai Maclin Biochemical Technology Co., Ltd (Shanghai, China). Hydroxypropyl trimethyl ammonium chloride chitosan (HACC) with a molecular weight of 800,000 was acquired from Jiaxing Core Bio-Tech Co., Ltd (Jiaxing, China). Unless otherwise stated, all chemicals were of analytical grade. *Escherichia coli* O157:H7 (*E. coli*, ATCC 25922) and *Bacillus subtilis* (*B. subtilis*, ATCC6633) were bought from Sigma-Aldrich Corp., St. Louis, MO, USA. Human umbilical vein endothelial cells (hUVECs) were purchased from the China Center for Type Culture Collection. A bicinchoninic acid (BCA) protein assay kit was purchased from Jiancheng Bioengineering Institute (Nanjing, China). Luria–Bertani broth (LB Broth), RPMI1640 culture medium, fetal bovine serum (FBS), Penicillin-Streptomycin solution (10 mg/mL) and Cell Counting Kit-8 (CCK-8) were purchased from Beijing LABLEAD Co., Ltd. (Beijing, China). Ultrapure water from a Milli-Q filtration system (Millipore Corp., Bedford, MA, USA) was used to prepare all solutions.

### 4.2. Preparation of Crosslinker-Free Bilayer Assembly Microspheres

**Figure 7:**
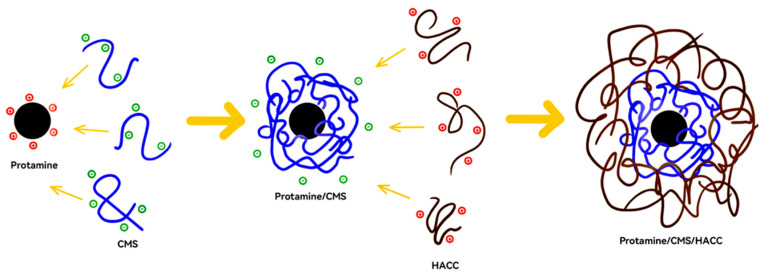
Schematic of the microspheres of bilayer (Protamine-CMS/HACC) assemblies synthesized through layer-by-layer (LbL) assembly.

Protamine-CMS/HACC was prepared through layer-by-layer (LbL) assembly based on the ionic interaction of polysaccharides (Figure 7). Zeta-potential measurements and encapsulation efficiency were used to assess the optimum assembly conditions. Typically, 0.1 g of protamine is dissolved in a solution with a pH 3.0 solution and then added dropwise to CMS solution at various mass ratios of 1/1, 1/2, 1/3, 1/4, and 1/6 under reaction temperatures ranging from 25 °C to 55 °C under stirring for 0.5 to 4 h. After centrifugation at 8000 rpm for 10 min, the precipitate was collected and washed with distilled water three times. After freezing for 12 h, white dry powders were obtained. Subsequently, 0.2 g of the above-obtained CMS-protamine microspheres was suspended in 1.6 mL of positively charged HACC solution (pH 4.0, 1 mg/mL) and incubated at 25 °C. After centrifugation at 6000 rpm for 3 min, the resulting HACC/CMS–protamine microspheres were collected and washed with distilled water and acetone. The assembly was freeze-dried for 12 h to obtain a yellow powder of double-layer-assembly microspheres.

The supernatant and washing liquids were collected for further determination of the non-encapsulated protamine content using a BCA assay. All experiments were performed in triplicate and the standard curve was

Y = 1.9952X − 0.0483 (R^2^ = 0.9901) (1)

The loading capacity (LC) and encapsulation efficiency (EE) were used to evaluate protamine assembly under different assembly conditions. They were calculated according to the following formula:

EE(%,*w*/*w*) = (W_(total protamine) − W_(free protamine))/W_(total protamine) × “100%” (2)

where W_(total protamine) is the initial amount of protamine; W_(free protamine) is the amount of unloaded protamine measured in the supernatant and washing solution.

### 4.3. Microsphere Characterization

The average particle size and zeta potentials were measured using a Nano-ZS Zetasizer dynamic light scattering (DLS) instrument (Malvern Instruments Ltd., Malvern, UK) at a concentration of 1 mg/mL, after filtering through a 0.22 μm membrane. The microstructure and surface morphology of the sample were characterized by a scanning electron microscope (SEM, ZEISS-ULTRA55; Hitachi Ltd., Tokyo, Japan) with a 3 kV accelerating voltage. IR analyses were conducted to qualitatively determine the surface functional groups using a Nicolet 5700 FTIR spectrometer (Thermo Fisher Scientific, Waltham, MA, USA) using the KBr-disk method in the range of 400–4000 cm^−1^. UV spectrophotometry was performed on a Nanodrop 2000 UV–vis spectrometer with a full-spectrum scan from 190 to 800 nm. The specific surface area (SSA) and pore structure were determined according to the Brunauer–Emmet–Teller (BET) model and the Barrett–Joyner–Halenda method. The surface area (SA) and porosity of the AC adsorption branches of the isotherms were analyzed using density functional theory (DFT).

A certain amount (approximately 0.2 g) of composite microspheres was immersed in distilled water at room temperature. After a period of time, the composite microspheres were removed and the excess solvent on the surface was removed quickly with absorbent paper and weighed. The swelling ratios were calculated as follows:

(3) $$Swelling\;ratio={(m}_{s}-m_{d})/m_{d}\times 100\%$$

where m_d_ and m_s_ are the weights of dry and wet film, respectively.

### 4.4. Antibacterial Test

Gram-negative bacteria *Escherichia coli* O157:H7 (*E. coli*, ATCC 25922) and *Bacillus subtilis* (*B. subtilis*, ATCC6633) were cultured for 12 h in a shaker at 37 °C. An amount of 1 mg of the samples was added to 1 mL of the bacterial suspensions (OD600 = 0.8) with a final concentration of 15 mg/mL; free protamine was also added as a control. After incubation at 37 °C for 4 h, 100 µL of 10-fold diluted suspensions from each test strain solution were spread onto agar plates and incubated for another 20 h at 37 °C. The bacterial colonies were counted and the colony number of bacteria without treatment was used as the control. The antibacterial efficiency was calculated as follows:

(4) $$Antibacterial\;rate\left(\%,\frac{cfu}{cfu}\right)=\frac{cell\;numbers\;of\;control-cell\;numbers\;of\;samples}{cell\;numbers\;of\;control}\times 100\%$$

### 4.5. In Vitro Cytotoxicity and Compatibility

The in vitro cytotoxicity was measured using a standard CCK8 assay. The hCMEC cells were seeded into a 96-well plate at a density of 5 × 10^3^ cells/well and incubated for 24 h until the cells adhered to the wall. The old culture medium was carefully sucked out and replaced with sterile treatment containing different concentration gradients (0, 10, 20, 40, 60, 80, 100) μg/mL) of the serum-free medium of nanoparticles; 5 multiple wells were set for each concentration. After co-culture for 24 h and 48 the old culture medium was carefully sucked out and washed twice with PBS, and 100 μL fresh medium was added. Under dark conditions, 10 μL CCK-8 solution was added, and then shaken slightly to mix it. After incubation in a CO_2_ incubator at 37 °C for 1 h, the absorbance value (OD value) of each hole at 450 nm was measured using a microplate reader (AMR-100, Hangzhou Aosheng Instrument Co., Ltd., Hangzhou, China).

### 4.6. In Vitro Hemostatic Evaluation

The whole blood used for hemostatic tests was collected from calves. Sodium citrate was added to the blood at a ratio of 1:9 (*v*/*v*) as the blood anticoagulant and then kept at 4 °C until further use. To evaluate the blood plasma clotting performance, whole blood (5 mL) was added to a centrifuge tube containing 5 mg microspheres. After incubation for 10 min, 25 mL of deionized water was carefully added without disturbing the clotted blood and then 200 μL of supernatant was taken from the bottles. The absorbance at 415 nm was measured using a microplate reader. The blood clotting test was carried out by comparing the relative absorbance of blood samples. The absorbance of 0.50 mL whole blood in 25 mL deionized water at 415 nm was assumed to be 100 as a reference value. All tests were duplicated three times. The blood clotting index (BCI) can be calculated according to the formula as

(5) $$BCI\left(\%\right)={OD}_{Sample}/{OD}_{blank}\times 100\%$$

### 4.7. Release Characteristics Characterization

The samples were added to phosphate-buffered saline (PBS) at pH 7.4 and placed in microcentrifuge tubes containing 1 mL of PBS at 28 °C. A sample solution with a concentration of 10 mg/mL was prepared and reacted under shaking conditions at 150 rpm. At different time points, the samples were centrifuged at 5000 rpm for 4 min, and then 0.2 mL of the supernatant was carefully collected and the fluorescence intensity was measured at 434 nm. After measurement, the 0.2 mL sample solution was returned to the system to obtain the total protein release at that time point. A standard curve of [concentration–slope] was constructed in the range of 10 μg to 1 mg (showing a linear relationship within this concentration range), and the concentration of unknown samples was interpolated based on the slope of each sample. The cumulative release of protamine over time was calculated using the following formula:

Cumulative release percentage (%) = (protamine supernatant/total protamine) × 100%

Here, the protamine supernatant represents the amount of protein released cumulatively at each time point, and the total protamine refers to the previously determined actual protein loading amount.

## Data Availability

The original contributions presented in this study are included in the article. Further inquiries can be directed to the corresponding author(s).
